# Unaligned connections or enlarging engagements? Tertiary education in developing countries and the implementation of the SDGs

**DOI:** 10.1007/s10734-020-00651-x

**Published:** 2020-12-04

**Authors:** Elaine Unterhalter, Colleen Howell

**Affiliations:** University College London, London, UK

**Keywords:** Tertiary education, Sustainable Development Goals (SDGs), Millennium Development Goals (MDGs)

## Abstract

Given that tertiary education (TE) is a sector often associated with exclusion, particularly in low- and middle-income countries (LMICs), where only a small proportion of the population gain access, how well placed is this sector to support the implementation of the SDGs? This article extends our reflections from a recent rigorous review of literature, published from 2010, which looked at the role of tertiary education in low- and lower-middle-income countries. The review noted the sparse literature on a range of development outcomes, with limited attention to some of the key themes of inclusion and sustainability associated with the SDGs. Many studies report on some form of limited connection between TE and development outcomes, also drawing attention to contextual conditions beyond TE that contribute to this. The article considers the reasons for these findings, and some of the difficulties of forming conclusions on a still limited base of research evidence. A second theme in the literature reviewed highlights that where TE establishes partnerships, engagements or cross-institutional alliances, joint and valuable learning in support of the SDGs ensues, enhancing practice and building institutions. Some of the implications of these findings for the positioning of TE in developing countries in the wake of COVID-19 are considered.

## Introduction

Many commentators critiqued the Millennium Development Goals (MDGs) for limited ambition with regard to education, which constrained capacity to achieve the full range of goals (Jones 2008; Unterhalter and Dorward 2013; Fredman Kuosmanen and Campbell 2016). In the debates about the successor policy framework, leading up to the adoption of the Sustainable Development Goals (SDGs), tertiary education (TE) was promoted both as a target in its own right and as a means of implementation for many other goals (Boni, Lopez-Fogues and Walker 2016; Unterhalter 2019; McCowan 2019). SDG 4 expresses a vision to ‘ensure inclusive and equitable quality education and promote lifelong learning opportunities for all’ with the target for SDG 4.3 ‘to ensure equal access for all women and men to affordable and quality technical, vocational and tertiary education, including university’ (United Nations 2015: 17). As studies of the synergies between SDGs show (Pradhan et al. 2017; Biggeri et al. 2019), SDG 4 has a high level of correlation with all the other SDGs. But studies done in this form looking at synthesising indicators, or trade-offs and synergies between goals and targets, do not tell us whether TE is effectively supporting other SDGs. A number of critiques of SDG 4.3 highlight how the formulation of the targets and the indicators do not engage with the contexts of realisation (McCowan 2019; Allais and Wedekind 2020). Given that TE is a sector often associated with exclusion, particularly in low- and middle-income countries (LMICs), where only a small proportion of the population gain access to this level of education (UNESCO 2020), can this sector productively contribute to the overall SDG vision?

*Agenda 2030*, which sets the policy framework for the SDGs, formulates goals in holistic and inclusive terms. The policy is implicitly oriented to changing any association of TE with processes that ignore deprivation and exacerbate the crises of climate change. The policy suggests that this sector needs to enhance concern for the many rather than an elite few:

We are resolved to free the human race from the tyranny of poverty and want and to heal and secure our planet. We are determined to take the bold and transformative steps which are urgently needed to shift the world onto a sustainable and resilient path. As we embark on this collective journey, we pledge that no one will be left behind. (UN 2015, Preamble).

There were many difficulties to achieving this vision even before the profound shocks associated with COVID-19, that have now been outlined in the 2020 SDG Report (UN 2020). Our own analysis of SDG4, working as part of a team documenting views regarding higher education and the public good in four African countries, assembled before the pandemic, suggested that the contributions of higher education to development and the creation of inclusive societies are enabled or constrained by contextual conditions. These may be understood as conditions of possibility within different countries (Allais et al. 2020; Unterhalter et al. 2019). These contingent and contextual issues raise questions as to how well positioned TE is in terms of policies, practices and systems to assist with the implementation of the SDGs, particularly in the wake of the COVID-19 pandemic.

In this article, we explore this concern through a consideration of the extent to which TE may be contributing to taking forward the vision of the SDGs in low- and lower-middle-income countries (LLMICs). We draw on and expand a rigorous review of literature, completed in 2020, which looked at the role of TE in development in LLMICs (Howell, Unterhalter and Oketch 2020). The study was concerned, not so much with questions of access and participation in TE, on which there is considerable literature (Schendel and McCowan 2016; Welch 2016; UNESCO 2020), but rather at some of the development outcomes of TE that might support more inclusive processes within TE and contribute to the realisation of the wider vision of the SDGs. The first part of this article describes the methods used in the rigorous review, discusses some of its conceptual underpinnings and summarises some findings from the body of literature surveyed and synthesised. The second part draws out some of the implications from the literature reviewed for assessing the capacity of TE in LLMICs to implement the SDG vision. Two themes in the literature reviewed are discussed. Firstly, we assess the nature of the literature that remarks on misconnection and misalignments between TE and particular development outcomes and what this might signal for SDG implementation. Secondly, we discuss literature that points to an enlarging engagement of TE, suggesting a widening range of partnerships in relation to SDG implementation. In conclusion, we consider some of the disruptions COVID-19 may have wrought on TE’s potential contribution to the realisation of the SDG vision, and consider what some of the implications are drawing on the literature reviewed.

### A rigorous review of literature on the role of tertiary education in development in LLMICs

The rigorous literature review mapped and synthesised evidence which analysed the relationship between TE and development in LLMICs. This study, which looked at literature published in English since 2010, was conceived partly as an update of the rigorous review of evidence in the same area undertaken by Oketch, McCowan and Schendel in 2014. The 2020 review took a broad view of TE and considered the role of TE in development as linked with processes that support a range of development outcomes. The work expanded the analysis of the 2014 study using a slightly broader conceptualisation of the core functions of TE and associating these with a slightly broader group of development outcomes than those outlined by Oketch, McCowan and Schendel (2014). The functions of TE delineated in the 2020 review comprised teaching and learning, research, innovation and engagement (Howell Unterhalter and Oketch 2020).

The framing for the 2020 study was influenced by our recent work on a project that looked at the public good role of higher education in four African countries, *Higher Education, Inequality and the Public Good (HEIPuG)* (Unterhalter et al. 2019).^1^ This study had alerted us to higher education’s public good role, implicit in the notion of development outcomes, as requiring conceptu alisations of ‘instrumental’ and ‘intrinsic’ dynamics (Ibid). These dynamics suggest that both monetary and non-monetary development outcomes associated with higher education may be viewed as the result of instrumental pathways, which link people, relationships, and institutions beyond a TE institution to enable particular development outcomes. What we view as ‘intrinsic’ pathways associated with public good or development outcomes, entail that change is enabled through processes, relationships and forms of engagements, which are located primarily within higher education institutions. These have indirect or complex connections to and influence on attitudes, practices, social networks, human well-being, and the functioning of institutions and structures associated with development outcomes. The HEIPuG study suggested that higher education may therefore provide ‘spaces’ that enable these relationships, perspectives or processes that can contribute to development, although these pathways might be complex and multi-faceted. Thus, instrumental or intrinsic orientations of higher education to development outcomes may be in the form of ‘straight’ cognitive gains, including increases in information, knowledge and understanding, or help build non-cognitive aspects, including processes that are affective, dialogic, equitable and concerned with well-being and sustainability. We were aware that this is an area of considerable debate in the field of higher education studies (e.g. Nussbaum 1998; Marginson 2016; Collini 2017; Willetts 2017; Mamdani 2019). For the purposes of our rigorous review, we regarded both the ‘instrumental’ and ‘intrinsic’ in the relationship between higher education, the public good and development outcomes as important to our thinking. We considered that this understanding of higher education could also be applied to the broader field of TE.^2^ For the review, we therefore developed a conceptual framework that allowed us to take the widest possible view of the instrumental and intrinsic ways in which the relationship between TE and development is enacted. This resulted in us identifying nine ‘economic’ and ‘non-economic’ development outcomes that we considered in our analysis of the literature.

The (HEIPuG) project had drawn our attention to the ways in which TE may function, sometimes directly and sometimes indirectly, to undermine positive normative outcomes associated with development (Unterhalter et al. 2019). For example, TE may be associated with the reproduction of social inequality and the political entrenchment of elites (Daloz 2018). The HEIPuG study highlighted how contextual factors enable or constrain public good outcomes of higher education and the conditions that make the realisation of such outcomes possible (Unterhalter et al. 2019). We therefore worked from the premise that particular conditions (operating at global, national, system, institutional, group and individual levels) enable or constrain the relationship between TE and development, taking the broadest possible understandings of both. Our framework for thinking about development was mainly influenced by the UNDP’s human development approach with a stress on improving people’s lives and expanding opportunities, rather than on increasing growth. It stresses building environments in which people have opportunities to improve well-being, and that we are able to ensure ‘an equitable, sustainable and stable planet’ (UNDP 2020). Our reflections on TE as contributing to building the environment for human developments drew on McCowan (2019), who presents an analysis of the developmental university, emphasising the multiple understandings and processes associated with development outcomes.

The conceptual frame that we used to map and analyse the literature for the rigorous review is captured in  the diagram below.

**Fig. 1:**
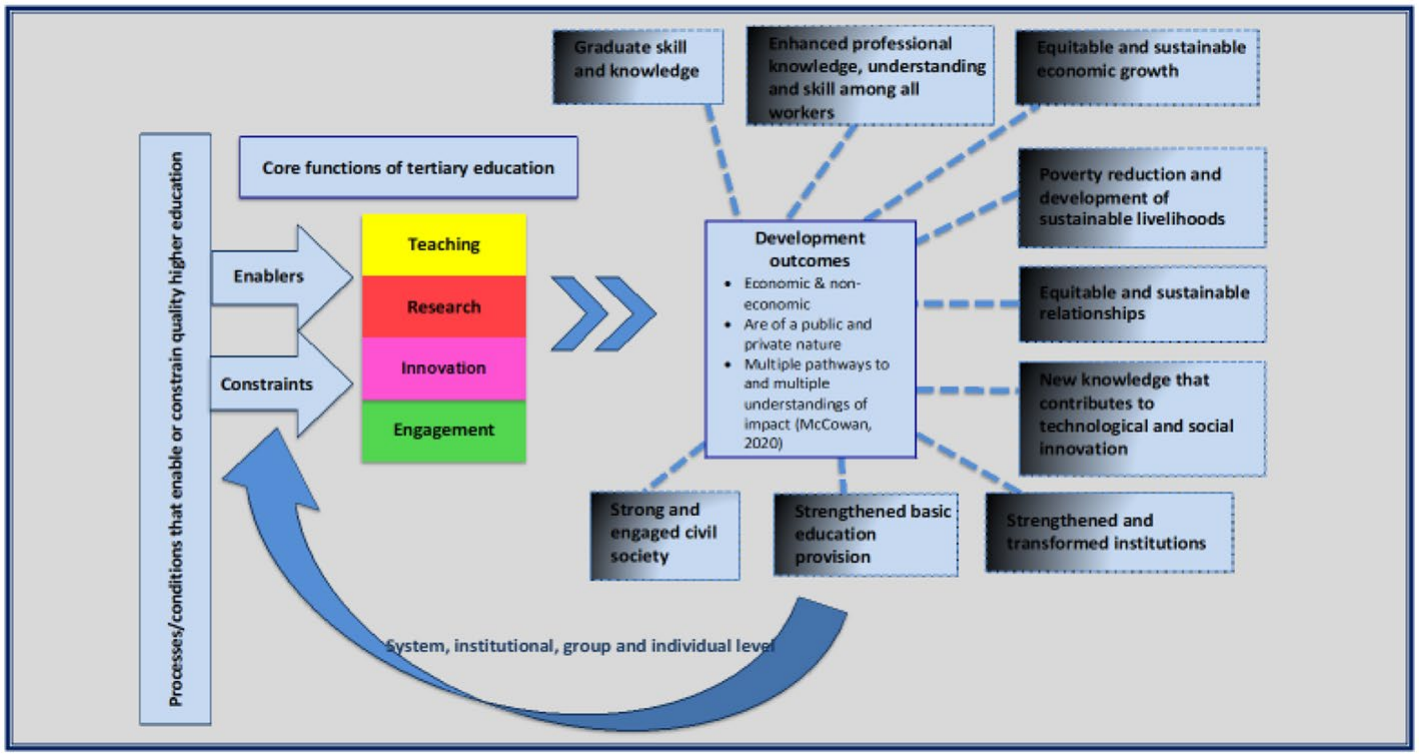
The diagram shows on the left-hand side that TE institutions, the people that work and study in them, and the systems that support them, are located in a context that will enable and/or constrain their contribution to development. These conditions of possibility are both historical and contemporary. They are largely structural and operate, as already noted, at system, institutional, group and individual levels, often enabling or constraining change in complex ways through TE’s core functions and the pathways from these leading to development outcomes. These pathways to change are captured through the central arrow and the smaller lines reaching out to each of the development outcomes. The diagram suggests through the dotted nature of these lines a relationship of change between TE and the outcomes that are directed towards them but may not cause them. The dotted lines also depict our assumption that the pathways to change from TE to development outcomes may be complex as well as simple and linear. The depiction of the development outcomes surrounded by a dotted border emphasises that they may overlap and the parameters between them are often fluid. The nine development outcomes are each presented as having a dark and light fill, emphasising that the outcome of the relationship between TE and development may be conceived of as normatively desirable, but in practice the change may be insufficiently or inadequately aligned to the development outcome, which we captured collectively as ‘misaligned’, or it may have negative consequences, leading to a mixture of implications for development

#### Figure 1 

While this thinking about the processes of linking TE to development outcomes was central to the conceptual frame we used for the review, it also has implications for thinking about the contribution of TE to the implementation of the SDGs. Thus, TE may contribute to a direct implementation of the SDGs through the changes to people, relationships and institutions enabled through ‘instrumental’ pathways, such as knowledge sharing, contributing to better health, poverty reduction, food security or technological change. On the other hand, ‘intrinsic’ pathways of TE may enable change towards the SDG vision indirectly through processes, relationships and forms of engagement, such as providing settings for critical review, convening multi-faceted partnerships with other institutions or networks, or through modelling and reproducing forms of engagement and practices that contribute to inclusive societies. These ‘instrumental’ and ‘intrinsic’ pathways may lead to one or more of the nine development outcomes, suggesting a range of ways in which TE may be associated with the implementation of the SDGs. However, TE may also be inadequately or poorly connected with such implementation, weakening its capacity to contribute to the realisation of Vision 2030.

The nine development outcomes that framed the rigorous literature review may be associated with a number of processes linked to the implementation the SDGs.

(i) Building graduate skill and knowledge
  This process and outcome is associated with the levels or kinds of knowledge and understanding, including technical expertise, and attributes or values that graduates may acquire through their study. Positive outcomes are associated with the learning gains of those who have participated in TE and the impact that this may have at the individual, family and broader societal level through, economic, cultural, social, health or political relationships. Examples are contributing to enhancing teachers’ training, knowledge and skill, thus meeting the target under SDG 4c for more trained teachers; or contributing to the training of health workers as outlined in the target SDG 3c, ‘Substantially increase health financing and the recruitment, development, training and retention of the health workforce in developing countries, especially in least developed countries and small island developing States’ (UN 2015). Poorly aligned outcomes could, for example, be associated with a mismatch between the knowledge, skill and values that teachers and health workers acquire through TE and the needs of the broader vision of SDG 4 on inclusive education, or the overall orientation of SDG 3 to ensuring healthy lives and promoting well being for all.
(ii) Enhancing knowledge, understanding and skill among all workers
  This outcome includes enhanced knowledge, understanding and skill, possibly gained through participating or working in TE or through the innovations and engagements associated with TE, that are important to serve society, contribute to its development, and enhance the well-being of its members. Examples are the knowledge, skill and understanding of teachers or health workers at all levels, not just the graduates covered in the outcome above. This category also includes enhancing knowledge for technicians, managers and public servants, administrators and manual workers employed inside and outside TE, who are not graduates, but who gain knowledge through initiatives linked to TE. For example, knowledge generated in TE may contribute to increasing food production as envisaged in SDG 2.3, a target that aims to:
  Double the agricultural productivity and incomes of small-scale food producers, in particular women, indigenous peoples, family farmers, pastoralists and fishers, including through secure and equal access to land, other productive resources and inputs, knowledge, financial services, markets and opportunities for value addition and non-farm employment (UN 2015).
  Positive outcomes here may demonstrate the ways in which TE contributes to this knowledge, understandings and skill, or may point to the ways in which access to relevant knowledge, skill and understanding to enhance food production has been restricted, is irrelevant or inadequately aligned to the concerns and needs of small scale producers.
(iii) Supporting economic growth
  This development outcome encompasses the ways in which TE contributes to the economic growth of a society, associated with increases in GDP, employment, levels of productivity and earnings. However, questions are raised concerning how equitable or inequitable such growth is and therefore in what ways TE may contribute to enhancing equity and sustainability. TE may contribute to addressing gender, regional, race, ethnic or disability inequities, but it may also deepen processes and practices that lead to the benefits of growth being lodged with small elites rather than being widely distributed. TE may also, through all of its core functions, address concerns for sustainable development and ‘green growth’, but it may also fail to address these. Thus, TE may contribute directly to the inclusive growth envisaged in the SDGs. For example, as outlined in SDG 8, which focuses on ‘inclusive and sustainable economic growth, full and productive employment and decent work for all’, and in SDG 10 on reducing inequality within and between countries (UN 2015). SDG 8 includes targets for ‘decent job creation’, equal pay for work of equal value, and decoupling economic growth from environmental degradation. The ways in which TE contributes to this would be covered by this development outcome. SDG 10 includes targets on increasing the income growth of the bottom 40% of a population and eliminating discriminatory laws, policies and practices. TE may contribute to an expansion of decent work and the elimination of inequalities or may deepen fault lines that leave people, communities and countries behind in the realisation of the 2030 vision.
(iv) Contributing to poverty reduction and development of sustainable livelihoods
  This development outcome is concerned with the connections or disconnections between TE and poverty reduction, a major concern in the SDG framework and the focus of SDG 1. Central here is how TE, through its programmes, may contribute to understanding and supporting the struggles of those contending with poverty and the creation of opportunities or pathways towards well-being, agency, equity and sustainability for these groups. Equally, it may point to ways that TE fails to do this. SDG 1, for example, has targets concerned with building the resilience of the poor or those in vulnerable situations and developing forms of social protection, areas that may be enhanced or overlooked by the functions of TE.
(v) Developing equitable relationships
  This outcome concerns equitable relationships within society and how TE may develop, influence and shape these relationships, articulated in many SDGs. For example, SDG 5, which deals with achieving gender equality and empowering all women and girls, and SDG 11, which deals with making cities and human settlements safe and inclusive. SDG 4.7 focuses on the importance of education that promotes gender equality, supports a human rights culture, and addresses sustainability. TE may contribute to either building equitable relationships as outlined in these or other goals and targets, or to destabilising or destroying such relationships, for example, through the promotion of ethnic divisions or the promotion of detached elites. In this way, TE may contribute to the equitable relationships envisaged through the SDGs, or it may deepen existing inequities, thus undermining the vision of the SDGs.
(vi) Supporting new knowledge that contributes to technological and social innovation
  This development outcome concerns the role TE plays in the creation of new knowledge through research and innovations, involving the application of knowledge to technological advancements, organisational development or social improvements. For example, SDG 9 is concerned with building infrastructure to support inclusive economic development and well-being and has targets on increasing access to ICT and upgrading science and technology in developing countries (UN 2015). SDG 13 is concerned with enhancing social innovation with regard to the impact of climate change, and SDG 10 has a target about changing approaches to migration in order that these are orderly, planned and safe. TE may contribute through its research and innovation functions to enhancing knowledge and skills to implement these kinds of targets. However, TE may also contribute to innovations that are linked with forms of harm, such as innovations underpinning mass surveillance, those used by extractive industries that cause environmental damage, or those involved in the development of destructive weapons systems.
(vii) Strengthening and transforming institutions
  This development outcome considers the role of TE in strengthening (or undermining) the ways in which institutions, such as those associated with education, health or decent work, are organised to support all sectors of society, deepen processes of inclusion, enhance accountability and transparency, and deliver knowledge, information and services. Also important here are initiatives that feed back into strengthening and transforming TE and other institutions, including developing enhanced capacity to implement the SDGs and take forward the 2030 vision. In the SDG framework, this development outcome is evident in a number of goals and targets, but in SDG 16, an aspiration is outlined for ‘effective, accountable and inclusive institutions at all levels’ with a stress on participation in decision making (UN 2015).
(viii)  Strengthening basic education provision
  This development outcome captures the relationship between TE and the basic education system directly, for example, through the training of teachers, or indirectly through collaborations and research, supporting, for example, curriculum development and quality enhancement outlined in SDG 4. Through these functions, TE may contribute to quality basic education that meets the needs of all. However, it may also provide training for teachers that is inadequate or misaligned to the targets in SDG 4.1 or 4.7, for example, or it may fail to maximise research and engagement opportunities that can strengthen basic education provision.
(ix) Supporting the development of strong and engaged civil society
  This development outcome is focused on the ways TE may support (or potentially undermine) the growth of an informed and engaged civil society concerned with supporting human rights, needs, well-being, capabilities and development. These processes are addressed in SDG 16, which stresses enhancing participation in institutions at all levels, supporting freedom of information, and developing cultures of peace building. TE may contribute to these goals through its ‘public intellectual’ role by supporting discussion and participation that creates the conditions for the realisation of these goals. But it may also restrict such discussion and engagement and undermine these relationships. This development outcome also draws attention to the critical role of TE in building necessary processes of reflection and critique around the SDGs and their implementation.
  Of these nine development outcomes, we consider (iii), (iv), (viii) as mainly oriented to instrumental pathways, (ix) as more oriented to a concern with ‘intrinsic’ processes through the public intellectual role of TE or as spaces of critique, and (i) and (ii), (v), (vi), (vii) mixing aspects of intrinsic and instrumental pathways.

### Methods for conducting the rigorous review

In conducting the rigorous literature review, we used the conceptual framework (Fig. 1) to develop research questions for the review and protocols for searching, coding and analysing the literature. We were aware that this process had limitations because of our development of the conceptualisation as slightly aloof researchers, working through the early phase of the coronavirus lockdown in London, and consulting primarily with an expert team, convened by the British Council, who had commissioned the study. However, we tried to mitigate this removal from everyday practice, by reviewing our conceptualisation through our continuing analysis of the data collected in 2019 in Ghana, Nigeria, Kenya and South Africa for the HEIPuG project, and through our discussions with members of that research team.

For the review, our first step was to search the literature using a range of search strings that took account of the various terms that may be used to describe tertiary education in different contexts. We aimed to review the literature published since 2010 from as wide a base of evidence as possible and searched the main relevant English language databases and online repositories and websites dealing with TE in LLMICs.^3^ This search and filtering process resulted in the identification of 2849 references, which we sifted further considering whether these were conceptually relevant to the concerns of the review. We recognised that establishing whether a contribution was conceptually relevant was a complex process. Our definition of conceptual relevance was based on whether the study explored in some way (explicitly or implicitly) the relationship between any facet, component or aspect of TE and one or more of the nine development outcomes noted in the conceptual framework. This relationship could be expressed in the form of a role, contribution, impact or in any other way it might be articulated, and may involve change at the individual, group, institution or system level. Screening the literature in this way resulted in the exclusion of 1313 references and a refined list of 1434 studies for more detailed review.

We examined these studies carefully assessing whether they provided evidence to advance ‘wider knowledge or understanding about policy, practice, or theory around the relationship of tertiary education delivered in LLMICs and development outcomes’ (Howell, Unterhalter and Oketch 2020, p27). We drew on analyses of assessing evidence (Gorard 2020; Gough Oliver and Thomas 2017) to determine if a study provided such evidence. These criteria required that a study must involve the application of some form of explicit research process, and, in addition, the work needed to meet at least one additional criterion, namely:

- The research process involves empirical or non-empirical research where there is clarity around the research methods used and there is a methodological fitness for purpose. Thus, the research design and methods used and the theoretical or disciplinary framework used are clearly described and discussed.
- The context of the study (historical, locational, political, economic, social or cultural) has been clearly described.
- The work has been subject to a process of peer review or the application of some form of external scrutiny.
- The study, even if small in scale, demonstrates credibility around the claims that are made by offering well-founded and plausible arguments about the significance/importance of the insights/findings generated (Howell, Unterhalter and Oketch 2020)

Applying these criteria, a final list of 170 studies was identified for detailed analysis. We noted at this stage that works included in the initial scan had largely been excluded from the final list because, although their titles and abstracts had suggested a link with development, on a closer reading the link between TE and development had not been clearly researched and was not supported by evidence. It thus appears that one of the major areas of growth of scholarship in the last 10 years has been description and investigation of the field of TE, but that far fewer works have actually researched links with development outcomes. This has implications for thinking about the implementation of the SDGs, as we discuss in the next section.

Table 1 summarises the distribution of the works included for detailed analysis highlighting the region of focus and the development outcome of main concern (coded as the 1st development outcome). It can be seen that the largest body of literature dealt with the relationship between TE and economic growth. The majority of these were studies by economists or economic historians. Two other substantial areas where scholarly work has been conducted concern graduate skill and knowledge and TE’s role in the creation (or not) of equitable relationships. Social research, sometimes emerging from sociological or anthropological studies was the most common disciplinary approach here.

**Table 1:** Distribution of evidence according to regional focus and main development outcome (*n* = 170)

Development outcome	Sub-Saharan Africa	South Asia	East Asia and Pacific	Latin America and Caribbean	Middle East and North Africa	Europe and Central Asia	Multi LLMIC regions	Total
Graduate skill and knowledge	12	5	4	0	3	1	0	25
Enhanced professional knowledge and skill among all workers	5	1	1	0	0	0	1	8
Economic growth	27	19	3	1	8	1	5	68
Poverty reduction and development of sustainable livelihoods	4	3	2	0	0	0	0	9
Equitable relationships	13	8	1	1	1	0	2	26
New knowledge that contributes to technological and social innovation	7	2	1	0	1	0	0	11
Strengthened and transformed institutions	2	1	0	0	0	0	1	4
Strengthened basic education provision	8	2	4	0	1	0	0	15
Strong and engaged civil society	4	0	2	0	1	0	1	8

Table 2 shows the core function of TE noted in the studies for final review. The majority of studies dealt with teaching and learning with only a small number focused on the research function. This limited engagement with the research function had also been noted by Oketch et al. (2014), suggesting severe limitations to the research capacity of TE in LLMICs, also noted in other works on this sector (Cloete 2011; Schneider and Maleka 2018). While this feature of the literature was not surprising, we were interested in the relatively large number of studies included in the review that dealt with TE engagement’s function. This was particularly evident in studies that highlighted the value of partnerships and TE, especially in the context of the ‘non-economic’ development outcomes. This highlights a number of issues with regard to implementing the SDGs that we discuss in the next section.

**Table 2:** The core functions of TE through which its role in development is enabled (1^st^ development outcome) (*n* > 170)

Core functions of TE	No. of studies
Teaching and learning	145
Research	14
Innovation	23
Engagement	40
Not clear	4

The evidence pointed at times to the involvement of more than one core function of TE. Source: Howell, Unterhalter and Oketch (2020, p. 24)

A central concern for us in the analysis of the evidence was to consider the ways in which the studies documented the pathway to change between one or more of TE’s core functions and the nine development outcomes, noting the direction and modality of the change process. Table 3 shows the nature of these pathways to change that we mapped, classified according to the main development outcome discussed in each work. The table shows whether the change noted was positive, misaligned or had features of both. It can be seen that the majority of studies of economic growth, poverty reduction, the enhancement of professional knowledge and skill, and a strengthened and engaged civil society, viewed the pathway to change as positive. However, for the other development outcomes, the majority of studies suggest pathways to change that were not well aligned with the development outcome or showed a mixture of positive, negative and poorly connected outcomes. The largest number of studies noted that development outcomes could be classified as having these mixed results. These studies often drew our attention to contextual issues as contributing to this misalignment, often by weakening the capacity of TE systems to contribute effectively to the development outcome.

**Table 3:** The nature of change between TE and the 1st development outcome (*n* = 170)

Development outcome	Positive	Misaligned	Both or mixed	Total (1st DO)
Graduate skill and knowledge	3	11	11	25
Enhanced professional knowledge and skill among all workers	4	1	3	8
Economic growth	31	13	20	64
Poverty reduction and development of sustainable livelihoods	5	1	3	9
Equitable relationships	5	7	14	26
New knowledge that contributes to technological and social innovation	1	4	6	11
Strengthened and transformed institutions	2	1	1	4
Strengthened basic education provision	4	7	4	15
Strong and engaged civil society	5	0	3	8
Totals	60	45	65	170

Source: Howell, Unterhalter and Oketch (2020, p. 25)

Another important area of concern was to consider the modality of the change process between TE and the development outcome. Table 4 shows the modality of the change process documented in the studies reviewed, showing whether the relationship between TE and the development outcome was presented as a linear or complex process. It can be seen that the majority of studies of economic growth present a linear pathway. For all the other development outcomes, it is more common to see complex pathways of change. Once again, we were made aware of the importance of context here, with these studies often highlighting the significance of contextual factors in contributing to this complexity.

**Table 4:** Modality of the change process between TE and the 1st development outcome (*n* = 170)

Development outcome	Linear	Complex
Graduate skill and knowledge	11	14
Enhanced professional knowledge and skill among all workers	2	6
Economic growth	41	23
Poverty reduction and development of sustainable livelihoods	4	5
Equitable and sustainable relationships	7	19
New knowledge that contributes to technological and social innovation	4	7
Strengthened and transformed institutions	1	3
Strengthened basic education provision	2	13
Strong and engaged civil society	0	8
Totals	72	98

Studies were coded as marking a linear change process when assertions were made about one facet of TE directly affecting a development outcome, such as enrolment in TE correlating with growth in GDP. Studies were coded as documenting a complex change process when the interactions of a large range of actors or relationships were analysed or the pathways to change were documented as multi-faceted. Source: Howell, Unterhalter and Oketch (2020, p. 25)

The tables presented above all provide a broad overview of the mapping of the literature in the review. It is not possible within the confines of this paper to discuss the more detailed findings in relation to each of the nine development outcomes or all of the cross cutting themes that emerged, which are dealt with in the full report.^4^ We turn now to expand that analysis, developing further some of the conclusions of the study and the implications for thinking about TE and the implementation of the SDGs in LLMICs.

### What do we know about how well positioned the tertiary education sector is in LLMICs to implement the SDGs?

The literature review yielded some insight with regard to what is known and not yet known about how well positioned the TE sector is in LLMICs with regard to implementing the SDGs. A finding, from the mapping of the literature included in the review, was that the largest proportion of studies was on the richest LLMICs. Thus, for South Asia, the largest number of studies were on India, and for Africa, on Kenya and Nigeria (Howell Unterhalter and Oketch 2020). The finding with regard to the relatively high volume of research from Kenya is consistent with the bibliometric analysis of the African Education Research Database (Mitchell, Rose and Asare 2020). This pattern of distribution highlights where research communities are located, where research money flows, where academics have time to conduct largely unfunded studies or how the use of English in research communities enhances publication prospects. It also shows how limited our knowledge resources remain with regard to the capacity of TE in the poorest countries to contribute to implementing the SDGs.

The review strengthens the concern that TE in LLMICs is located in a network of relationships that lean towards the interests, debates, and concerns of high-income countries, where the major relationships associated with publication, disciplinary prestige and grant disbursement are located. The majority of money is invested in universities in the Global North, where many students from LLMICs desire to study, and where research capacity is considerable. This magnetic pull of the Global North with regard to TE as a sector, runs the danger of orientating teaching and learning, research, innovation, and networks away from local engagement, because the agenda for all these facets of TE, crucial for the implementation of the SDGs, are largely set outside a particular LLMIC (Unterhalter and Carpentier 2010; Ilieva Beck and Waterstone 2014; Walker and Martinez-Vargas 2020).

From the literature review, the biggest body of evidence relates to aggregate economic growth or the earnings of graduates (see Table 1), but far fewer studies look at the themes of decent work or contribution to the income of the bottom 40%, as suggested by targets in SDG 8 or 16. From this review, there are only a small number of studies published in English that note the engagements of TE with poverty reduction and innovation, key targets in the SDG framework. Thus, the research on how TE may contribute to the implementation of the SDGs in poorer contexts is still sparse. We noted an enormous job to be done building research capacity, research environments, and supporting investigation of the view from below, whether this is documenting more extensively TE in the poorest countries, investigating the relationships of TE with non-elite groups, or researching interventions that seek to reduce, rather than widen economic or social inequalities. As all these themes connect with the process of implementing the SDGs, we concluded that the implication is not less TE, but more TE, with better support to connect teaching and learning to the research, innovation and engagement pillars of TE and the holistic SDG vision. These conclusions apply both to university education and to technical and vocational education and training (TVET).

One of the central conclusions reached in the review was that TE institutions in LLMICs are largely not managing to fully realise their potential with regard to their role in development, looking across a wide range of economic and non-economic development outcomes. Thus, for example, implementing SDG 4.3, only with an eye to student access without attending to how graduate knowledge and skill and other key facets of the TE sector are deployed, is to miss a huge opportunity, especially towards strengthening the capacity of TE in LLMICs. This requires understanding TE’s role in development as a two-way process, between TE and a range of partners and role players across a wide spectrum of policies and practices that enhance communication and collaboration. These relationships may be different for different forms of TE, but the need to build connection is important. With regard to thinking about this in relation to the SDG project, there is a need to understand some of the reasons for the limitation on the capacity of TE to deliver, that the review has suggested.

### Tertiary education and the implementation of the SDGs: understanding poor alignment and expanding engagement

Two themes emerged from the rigorous review of the literature that are particularly salient to considering the role and capacity of TE to support the implementation of the SDGs. The first concerns the weak alignment that was often evident between TE and a number of development outcomes (see Table 3). This finding suggests that TE may be constrained in its support to any substantive SDG implementation through weaknesses within TE systems, inefficiencies, or understandings that do not sufficiently consider the SDGs in context. Such trends may be associated with efforts that are aimed at growing the TE sector for its own sake or with the impact of the global inequalities across the sector discussed above. A second theme, which builds from the body of work on the engagement function of TE, suggests that when TE is engaged with local needs and complexities, and connects these with wider concerns, this gives promise of a considerable contribution to the implementation of the SDGs.

These two orientations may appear to contradict each other, but our discussion illustrates that both processes may be happening at the same time, but not for the same reasons. The first theme suggests that TE is not yet well enough placed to support the implementation of the SDGs, because it is not adequately producing appropriate skills and knowledge, or it is not undertaking research that is needed for their effective implementation. But the reasons why this theme is prominent in the literature reviewed needs some thought. The second theme suggests that, where important groundwork has been laid by TE to take forward partnerships, crucial for the implementation of the SDGs, this is particularly generative for both TE and a range of development outcomes. However, many initiatives in this area are fragile and need deepening through practice and further research.

As already reported, weak alignment between TE and development outcomes was noted in a number of studies and appears to suggest that TE in LLMICs may not currently be well placed or have sufficient capacity to effectively support the implementation of the SDGs. As Table 3 shows, poor alignment was noted in many studies in relation to how TE connected with development outcomes. For example, studies suggested a perceived ‘mismatch’ between the skills nurtured in TE and the demands of the labour market (Amadi and Ememe 2013; Jonbekova 2015; Moono and Rankin 2013; Uzair-ul-Hassan and Zahida 2013), the requirements of employers (Agabi et al. 2012; Alagumalai, Kadambi and Appaji 2019; Kintu et al. 2019) and students’ adaptability to the world of twenty-first century work, especially with regard to ‘non-technical’ or ‘soft skills’ (Esthim 2017; Gokuladas 2010). A number of these studies concluded that perceived curriculum weaknesses in TE derived from insufficient collaboration between TE institutions and employers in the design and development of courses (Agabi et al. 2012; Amadi and Ememe 2013; Esthim 2017; Moono and Rankin 2013). However, there is an issue as to how much this weak alignment is associated only with the relationships within TE and how much this is because the sector is located within a particular political and socio-economic context or set of historically inflected relationships. A number of studies drew attention to processes outside TE that contributed to the misconnection of graduate knowledge with development outcomes. For example, Kleibert (2015) reported how large foreign investors employed university graduates in low skill work in the Philippines. Islam et al. (2019) reported in Bangladesh how gender relationships made it difficult for female graduate entrepreneurs to put their skills into action. These works suggest that the mismatch between graduates and employment and productivity demands are not just about what is or is not learned in TE, but rather a complex relationship associated with the strategies of firms and existing social relationships, of gender or ethnicity, which come to be reproduced in a misaligned or negative direction of change between TE, economic growth and equity. The implications of the body of literature we categorised as misalignment, may suggest that TE in LLMICs produces graduates with knowledge that does not always meet the requirements of the work place, or has limitations with regard to productivity. An alternative conclusion is that it is relationships and conditions outside TE with regard to decent work, gender equity, and responses to climate change, that have to change, as the SDG targets indicate.

We recognised that the dominance of this theme of misalignment may suggest that institutions are remote from the real needs of countries and thus weakening their capacity deliver on the SDGs agenda. But greater nuance is needed before confirming this conclusion. The literature discussed through the rigorous review may have its own particular focal points. Research conducted and reported may concentrate on ‘black swans’, precisely because they are not typical, and not what is expected. Thus, while it is routine to assume that TE produces graduates to fill positions needed in a society and in line with the implementation of many SDG targets, this everyday process, or widespread presence of ‘white swans’, does not catch the attention of researchers. What may become a feature of investigation is not the routine or expected, but what is surprising. Thus, what is noteworthy for research is that TE may not be producing appropriate graduates. Thus, it is the relatively rare presence of ‘black swans’ that generates inquiry. Given that the literature on TE and development outcomes in LLMICs is still relatively small, that many findings in these countries are not triangulated, and that few studies around many of the development outcomes are published in English (see Table 1), it is plausible that the misalignment and misconnection pointed to through the studies between TE and development outcomes may be overstated through the methods associated with a rigorous review, which looks at what is published, rather than what exists on the ground.

Bearing these caveats in mind, however, it is still important to explore some implications of what are presented as the poor alignments, misconnections and miscommunications for the capacity of TE to implement the SDGs. In a number of the studies where poor alignment was noted, the evidence directed attention towards the sector itself and perceived weaknesses within it that suggest failures by TE institutions to adequately understand and respond to the complexities of local needs. A number of studies suggested that the curriculum offered by TE programmes was failing to appreciate what is needed in practice. This included forms of classroom practice needed by teachers in basic education (Al Amin and Greenwood 2018) or curriculum and assessment practices that do not generate the ‘transformative socio-economic knowledge’ required by many LLMICs (Niyibizi et al. 2018). Other studies suggested that disciplinary approaches and practices in TE fail to draw on the knowledge, understanding and experiences of important stakeholders such as employers (Agabi et al. 2012; Amadi and Ememe 2013; Esthim 2017; Kintu 2019; Moono and Rankin 2013), or communities (Mbah 2015) located outside TE institutions. Some studies argued that TE teaching was not drawing adequately on global debates important for students’ and future professionals (Amutuhaire 2013). Collectively, these findings suggest that TE’s contribution to effective implementation of the SDGs requires careful consideration of how teaching, learning, research and engagement connect to enable good understanding of local contexts.

The success of TE systems in LLMICs in expanding engagements through different forms of partnership provided particularly useful examples of roles that could be performed by TE in implementing the SDGs. A number of studies spoke to the value and importance of partnerships within TE and between TE and other role players. This was evident in the literature on a number of the development outcomes but was particularly noted through the review of studies on the strengthening and transformation of institutions. This process we concluded from our synthesis of the literature ‘is strongly enabled through its [TE’s] participation within collaborative relationships and partnerships that are facilitated through its teaching and learning and engagement functions’ (Howell, Unterhalter and Oketch 2020, p 5). These included the strengthening of TE institutions themselves through partnerships or networks with other TE institutions or research bodies (Cloete, Bailey and Pillay, 2011; Allen 2014; Johnson et al. 2011; Amare 2017; McNae and Vali 2015), with different levels or structures of government (Magara et al. 2011; Situmorang et al. 2018) and with employers or industry partners (Kirby 2019). Other examples noted the involvement of students in internships with community organisations involving knowledge transfer (Magara et al. 2011) and the engagement of TE with local economic development strategies (Fongwa and Wangene-Ouma 2015).

This suggests that TE’s role in implementing the SDGs is enabled through participation in collaborative relationships and processes. But a number of studies also drew attention to how vulnerable these relationships and forms of engagement are, requiring important processes to maintain these links (Collins 2012; Luescher et al. 2011) and developing the capacity for TE systems and institutions to sustain collaborations under difficult conditions (Cloete et al. 2011; Kirby 2019). Ensuring that these collaborations, especially where they involve North–South partnerships or engagements with vulnerable groups, remain relevant and responsive to local contexts (Allen 2014; Ramos et al. 2012) and equitable (Thomas 2019), is also a key concern.

### Implementing the SDGs through tertiary education after the coronavirus epidemic

The rigorous review we reflected on in this article was completed in the early phases of the COVID-19 pandemic, and the literature included for synthesis did not therefore report on the political, economic and educational effects of the coronavirus. The SDG report (UN 2020a) shows how the effects of the pandemic have threatened many areas of work associated with the SDGs. But the effects of the virus and the political and economic actions associated with the response have been uneven. Some of the worst effects of the shutdown and the economic contraction have been felt by the poorest. This report noted how progress around the provision of quality education for all had been too slow before the virus, and education had been very unevenly delivered during the pandemic. Although no comment is made in the report on TE, other studies note how economic contraction may limit the resources for students to pay fees, and how limits on mobility may slow research agendas (Chan 2020). In many of the areas associated with the development outcomes considered in the review and in this paper, which constitute important areas of partnership for TE, such as expanding basic education provision or addressing poverty, the UN report notes that progress has slowed (UN 2020a).

A review of scenarios with regard to implementing the SDGs was carried out in 2020 for the July meeting of the High Level Political Forum on the SDGs (UN 2020b). This outlined setbacks, uncertainties and opportunities for the SDG agenda linked with supporting universal healthcare, robust social protection, more forceful action on climate change, better protection of land, water and biodiversity, and greater recognition of sustainable production and consumption. None of these scenarios deal explicitly with education, although the conclusion of the meeting report draws out the significance of better governance and partnerships to achieve the SDGs. This theme, as discussed above, also emerged from our rigorous review of literature as one of the key ways in which TE can build engagements for implementation of the SDGs. It appears to us that strengthening TE in LLMICs is not only a scenario for realising the SDGs but is an important development initiative in its own right.

TE has not been able to prevent the current terrible epidemic, and as a sector, it cannot forestall future epidemics or disasters. But the literature we have reviewed highlights that TE has an important role to play in helping people prepare better and support each other through periods of hardship, loss and uncertainty, such as the COVID-19 crisis. The SDG framework points to many areas to focus this support. To enhance this process the literature we have reviewed suggests that there is a need to understand how the work of teachers, learners, researchers or practitioners in TE in LLMICs may be weakly or inadequately aligned to the implementation of the SDG agenda, and, if this is the case, what reasons account for this. Knowledge, insight and research into these processes can help support better actions to make and sustain the connections needed between people, places, disciplines, and forms of practice enjoined by the SDGs. Building on the work already done for enlarging engagements of TE seems an important contribution that can be made to the vision outlined in the Foreword to the SDG Review (UN 2020a) by the UN Secretary General, Antonio Guterres. He has called for ‘renewed ambition, mobilization, leadership and collective action’ not just to confront COVID 19, but to deliver on the SDGs. His views are echoed by the UN Under Secretary General for Economic and Social Affairs, whose message in the same volume is to hold fast to the convictions outlined in the SDG programme and build back better. This process we consider requires enhancing connections already made by those working in and with TE, and using these connections to re-imagine a sector better aligned to implement the SDG vision.

## Footnotes

1 This project was funded jointly by the Economic and Social Research Council (ESRC) Newton Fund and the National Research Foundation (NRF), South Africa, Grant Number ES/P002498/1 and UCL GCRF funds for a project on Decolonizing teacher education.

2 The review used a working definition of TE as systems and institutions providing formal education beyond secondary school. This encompassed university education, post-secondary technical, vocational education and training provided through institutions offering formal qualifications, professional training leading to a professional or vocational qualification such as that offered through business and medical schools, polytechnics and technical colleges, teacher-training colleges and two-year further education institutions.

3 Main databases and websites searched: ERIC (ProQuest or EBSCO); Social Sciences Citation index; International Bibliography of the Social Sciences; DFID; World Bank (JOLIS for World Bank and IMF literature); UNESCO; African Journals Online; Asia Journals Online; Latin America Journals Online; International Development Research Centre; CODESRIA; African Education Research Database (AERD); International Association of Universities; Association of African Universities; Association of Commonwealth Universities; HERANA—Higher Education Research and Advocacy Network in Africa; Centre for Higher Education Transformation (CHET) A preliminary search of smaller websites of development co-operation partners working in LLMICs also took place.

4 Available at https://www.britishcouncil.org/research-policy-insight/research-reports/tertiary-education-development
